# Structural Phase Transitions in *closo*-Dicarbadodecaboranes C_2_B_10_H_12_

**DOI:** 10.1021/acs.inorgchem.1c04022

**Published:** 2022-04-01

**Authors:** Matteo Brighi, Fabrizio Murgia, Zbigniew Łodziana, Radovan Černý

**Affiliations:** † Department of Quantum Matter Physics, Laboratory of Crystallography, University of Geneva, Quai Ernest-Ansermet 24, CH-1211 Geneva, Switzerland; ‡ Polish Academy of Sciences, Institute of Nuclear Physics, ul. Radzikowskiego 152, 31-342 Krakow, Poland

## Abstract

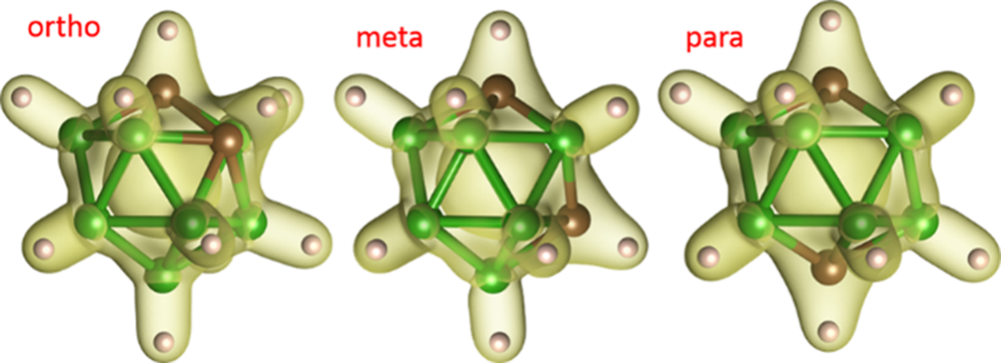

The crystal structures of three thermal polymorphs (I, II, and III) for each isomer of *closo*-dicarbadodecaboranes C_2_B_10_H_12_ (*ortho*, *meta,* and *para*) have been determined by combining synchrotron radiation X-ray powder diffraction and density functional theory calculations. The structures are in agreement with previous calorimetric and spectroscopic studies. The difference between rotatory phases (plastic crystals) I and II lies in isotropic rotations in the former and anisotropic rotations of the icosahedral clusters in the latter. Phase I is the cubic close packing (*ccp*) of rotating *closo*-molecules C_2_B_10_H_12_ in the space group *Fm*3̅. Phase II is the *ccp* of rotating *closo*-molecules C_2_B_10_H_12_ in the cubic space group *Pa*3̅. The preferred rotational axis in II varies with the isomer. The ordered phases III are orthorhombic (*meta*) or monoclinic (*ortho* and *para*) deformations of the cubic unit cell of the disordered phases I and II. The ordering in the phase III of the *ortho*-isomer carrying the biggest electrical dipole moment creates a twofold superstructure *w.r.t.* the cubic unit cell. The thermal polymorphism for C_2_B_10_H_12_ and related metal salts can be explained by division of the cohesive intercluster interactions into two categories (i) dispersive cohesive interaction with additional Coulombic components in the metal salts and (ii) anisotropic local interaction resulting from nonuniform charge distribution around icosahedral clusters. The local interactions are averaged out by thermally activated cluster dynamics (rotations and rotational jumps) which effectively increase the symmetry of the cluster. The C_2_B_10_H_12_ molecules resist at least as well as the CB_11_H_12_^–^ anion to the oxidation, and both clusters form easily a mixed compound. This allows designing solid electrolytes such as Na_*x*_(CB_11_H_12_)_*x*_(C_2_B_10_H_12_)_1**–***x*_, where the cation content may be varied and the temperature of transition into the disordered conducting phase is decreased.

## Abstract 2

The crystal structures of the three thermal polymorphs in the three isomers of *closo*-dicarbadodecaborane C_2_B_10_H_12_ are presented. The difference between rotatory phases I and II lies in isotropic rotations in the former and anisotropic rotations of the icosahedral clusters in the latter, both crystallizing with a cubic close packing of rotating *closo*-molecules C_2_B_10_H_12_. The ordered phases III are orthorhombic or monoclinic deformations of the cubic unit cell of the disordered phases I and II.

## Introduction

Carboranes are molecular polyhedral boron-carbon clusters C_*x*_B_*y*_H_*z*_ that are stabilized by electron-delocalized covalent bonding in the skeletal framework.^1^ They have been discussed in the classic paper of Lipscomb and Hoffmann^2^ before reports on their synthesis. The discussion focused on the extremely stable icosahedral cluster, a 12-vertex, 20-sided polyhedron, in the form of dianion B_12_H_12_^2–^, monoanion CB_11_H_12_^–^, and neutral C_2_B_10_H_12_. Icosahedral B_12_ clusters are present in all forms of elemental boron and in some metal borides.^3^ The first icosahedral carboranes had actually been prepared in industrial laboratories in the 1950s, although not reported in the literature until late 1963. They have been obtained in an effort to synthesize boron-based aircraft and rocket fuels that could exploit the much higher energies generated by combustion of boron hydrides compared to hydrocarbons.^4^ Nowadays, carboranes and their derivatives are extensively used in organic syntheses, medicine, nanoscale engineering, catalysis, metal recovery from radioactive waste, and a number of other areas.^1^ Additionally, carboranes are also effective building blocks in liquid crystals.^5^

**Figure 1:**
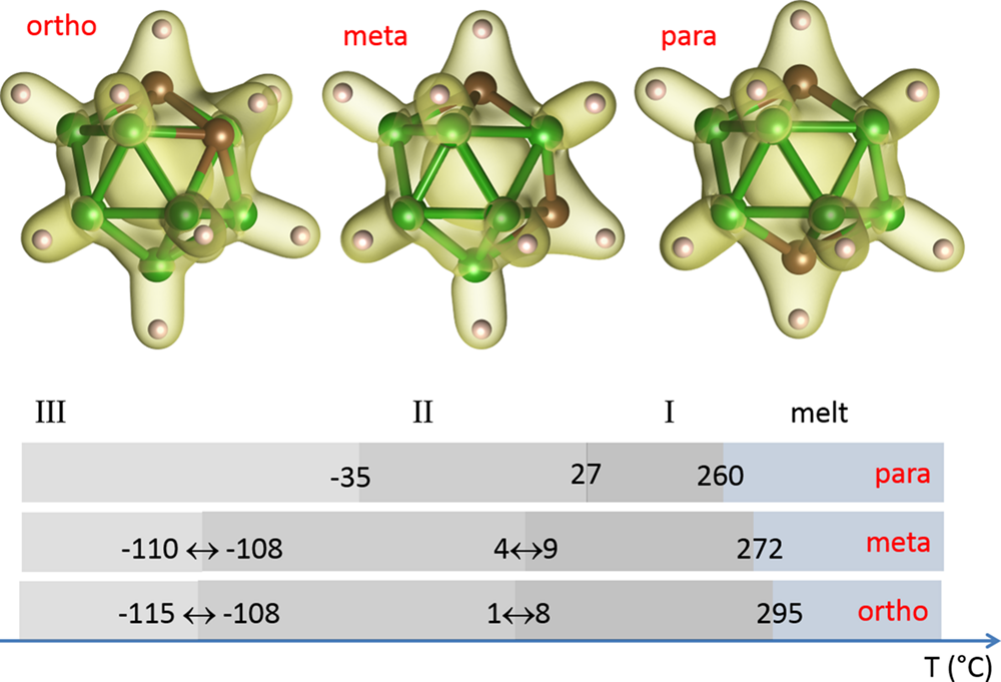
Molecular structure of C_2_B_10_H_12_ isomers with charge density painted at 0.09 e Å^–3^. Molecular symmetry: C_2v_ (2*mm*) for *ortho* and *meta* and D_5d_ (5̅2/m) for *para* (top). The temperature range of phase transitions and melting temperature detected in solids of three isomers (phase labeling according to ref (10)) with transition temperatures compiled from refs (10−15) and melting temperatures from ref (16) (bottom).

The icosahedral carborane molecule C_2_B_10_H_12_ (*closo*-dicarbadodecaborane) is known to exist in three isomeric forms: 1,2-C_2_B_10_H_12_, 1,7-C_2_B_10_H_12_, and 1,12-C_2_B_10_H_12_ called here *ortho*-carborane (*o*-), *meta*-carborane (*m*-), and *para*-carborane (*p*-), respectively (Figure 1). Their molecular structures in the gas phase have been determined from electron diffraction studies,^6−8^ thus providing an associated electrical dipole moment of 4.09, 2.58, and 0 D for the *o*-, *m*-, and *p*-isomers, respectively, as calculated in our work. The determination of the molecular structures in the solid state has been complicated by the important molecule dynamics at room temperature (*rt*), and it was determined first for the *o*-isomer by “taming” the disorder by means of cocrystallization with hexamethylphosphoramide.^9^

Solid C_2_B_10_H_12_ is a white powder under ambient conditions with high partial pressure, and therefore, sublimation from the solid state occurs before melting in open systems. The melting points have been determined in sealed tubes as 295, 272, and 260 °C for the *o-*, *m-*, and *p*-isomers, respectively.^16^ A face-centered cubic (*fcc*) cell with *a* = 9.86 Å has been suggested for the crystal structures of the *o*- and *m*-isomers under ambient conditions from X-ray powder diffraction.^11,17,18^ Further structural characterization provided controversial results, coherent at least in the existence of three temperature polymorphs for each isomer (Figure 1) with the exception of *o*-C_2_B_10_H_12_ where the presence of a fourth polymorph has been claimed between 1 and 22 °C.^18^ As we will show, the controversy of diffraction studies (leading to different structural models) lies in weak diffraction peaks and, at least for the *o*-isomorph on the temperature history of the sample and the cooling rate.^10^ The crystalline C_2_B_10_H_12_ is bound by weak dispersive interactions, and a fragile molecular ordering can be easily perturbed by rotational excitations even at such low temperatures as −100 °C.

In addition, at 470 °C the slow isomerization of *o*-carborane to *m*-carborane is observed. The latter isomer is converted, with low yield, to the *p*-carborane at 615 °C and undergoes decomposition at about 630 °C (see ref (11)).

The calorimetric, nuclear magnetic resonance (NMR), infrared (IR), Raman, and dielectric spectroscopic studies provided a coherent image of the polymorphic phase transitions between the rotatory phases (plastic crystals I and II) and ordered crystals (III):^10−14,19,20^

*Phases I&II*: The reorientation of the icosahedral molecules in phase I was found to be isotropic in the solids of all three isomers. While decreasing the temperature, the dynamics becomes more and more anisotropic in phase II for the *o*- and *m*-isomers. This is related to the decrease of the reorientation jump frequency along the C_3_ and C_5_ symmetry axes of the icosahedron leaving prominently rotation along the C_2_ axis, which is parallel to their respective dipole moments. The reorientation in the *p*-isomer has been observed to be isotropic in phases I and II according to ref (13), while anisotropic according to ref (19), preferring the C_5_ axis in phase II.

*Phases II&III*: The reorientation in phase III is considerably slower, pointing to an ordered phase in all three isomers with reorientation between symmetrically equivalent positions, that is, for the *p*-isomer rotation along a C_5_ axis containing both carbon atoms. The hydrogen–hydrogen interaction has been suggested as ordering force in the phase III, partly offset by dipole–dipole interaction in the *o*-isomer. The behavior of the phase transition II–III in the *o*-isomer depends on the cooling rate. Indeed, by fast cooling (10 K min^–1^) a metastable phase IIa, claimed “glassy”, is stabilized. This can be further transformed to a fully ordered crystalline phase by very slow cooling (0.1 K min^–1^).^10^ On heating the crystalline phase III, it transforms directly to the phase II.

We have been motivated to study the structures and phase transitions in the C_2_B_10_H_12_ isomers as a model case for interaction between icosahedral clusters in hydridoborate salts of lithium and sodium, technologically important as solid electrolytes for ion-batteries.^21^ Mixing of a neutral C_2_B_10_H_12_ molecule with an anion having the same icosahedral shape, that is, B_12_H_12_^2–^ or CB_11_H_12_^–^, stabilizes various packing of the boron clusters with a varying number of cations, Li^+^ or Na^+^, in the structure (work under progress). In this work, we will confirm by temperature-dependent X-ray powder diffraction, differential scanning calorimetry (DSC), and ab initio calculations the existence of three temperature polymorphs for each isomer and present their crystal structures resolving the ambiguity related to phase III. The origin of the metastable phase observed at low temperatures in the *o*-isomer is discussed as well. We will also provide insight into the electrochemical stability of *m*-C_2_B_10_H_12_ studied on its mixture with NaCB_11_H_12_ resulting in an Na^+^ conductor.

## Experimental Section

### Synthesis

The *o*- and *p*-carboranes C_2_B_10_H_12_ were purchased from Katchem Ltd. and *m*-carborane at abcr AG (purity ≥98%). A NaCB_11_H_12_/*m*-C_2_B_10_H_12_ mixture in a 1:1 molar ratio was prepared by mechanochemistry using a planetary mill Fritsch P7 at 500 rpm for 2 h (2 min milling, 2 min break, 30 cycles). The electrochemical test was carried out on a pelletized sample, by pressing the powder in a hydraulic uniaxial press, with pressures ranging from 100 to 550 MPa.

### Differential Scanning Calorimetry

DSC measurements were performed in the Department of Inorganic and Analytic Chemistry of the University of Geneva, using a Mettler-Toledo calorimeter, aluminum crucibles, and protective nitrogen flow (20 mL min^–1^).

### Synchrotron Radiation X-Ray Powder Diffraction (SR-XPD)

The crystal structures of the solid C_2_B_10_H_12_ isomers were studied by means of temperature-dependent SR-XPD, and the data were collected at Swiss Norwegian Beamlines BM01 (ESRF) with the Dectris Pilatus M2 detector and the wavelengths of 0.73990 and 0.64113 Å, calibrated with the NIST SRM640c silicon standard, in the temperature range of −150 < *T* < 200 °C. For all measurements, the samples were sealed into borosilicate capillaries of 0.5 mm diameter (under an argon atmosphere), which were spun during data acquisition. The temperature was controlled with a Cryostream 700 (Oxford Cryosystems) using a cooling and heating rate of 10 K/min. The 2D images were integrated and treated with the locally written program Bubble.

Temperature-dependent SR-XPD data with a very slow cooling rate (10 K/min from *rt* down to −98 °C, held for 12 h and then 0.1 K min^–1^ down to −105 °C, protocol according to ref (10)) were collected on a Panalytical Empyrean diffractometer in capillary mode (CuK_α_ radiation and Pixcel linear detector) for the *o*-isomer.

The crystal structures were solved ab initio using the software FOX^22^ and refined with the Rietveld method using TOPAS.^23^ The *closo*-molecule C_2_B_10_H_12_, was modeled as a rigid body with an ideal icosahedral shape and with corresponding B(C)-H and B-B(C) distances. All structural drawings were done with programs VESTA^24^ and DIAMOND.^25^

### Ab Initio Calculations

The calculations were performed within the density functional theory (DFT) method with plane wave basis sets as implemented in the Vienna ab initio simulation package.^26^ The parameters were as follows: cut-off energy for the basis set expansion 700 eV, the *k*-point sampling density *k*·a > 25, and the convergence criteria for electronic degrees of freedom 10^–6^ eV Å^–1^, for the structural relaxations in the conjugated gradient method with a convergence of 10^–2^ eV Å^–1^, projected augmented wave potentials^27^ are applied for atoms with the electronic configuration 2*s*^2^2*p*^1^ for B, 2*s*^2^2*p*^2^ for C, and 1*s*^1^ for H. The Perdew, Burke, Ernzerhof exchange-correlation functional^28^ with dispersion interactions^29^ that are important for aromatic boranes and carboranes was used.^30^ The calculations for isolated molecules were performed in a cubic box with an edge of 17 Å. The gamma point vibrations and dielectric properties are calculated within the linear response method.^31^ Barriers for molecular rotations were calculated with the nudged elastic band method.^32^

### Electrochemistry

Cyclic voltammetry (CV) was performed in a PTFE Swagelok cell, with a pellet dimension of 6 mm diameter and 0.5 mm thickness. A polished, hand scratched, sodium (Sigma-Aldrich, ACS reagent grade) foil was punched on a 5 mm disk (thickness < 0.1 mm) and was used as a self-reference electrode. Glassy carbon (Sigradur 180 μm thickness, purchased at HTW) was selected as a working electrode. To increase the active surface allowing the detection of small electrochemical events, the pellet side facing the working electrode was composed of a mixture of graphite (EC-600JD) and sample, following the protocol reported in ref (33). A close-to-equilibrium slow sweeping rate (20 μV s^–1^) was adopted to allow the detection of small surface processes. All the sample manipulations described in the Experimental Section were carried out in an argon-filled glove box (H_2_O and O_2_ < 0.1 ppm).

## Results

**Figure 2:**
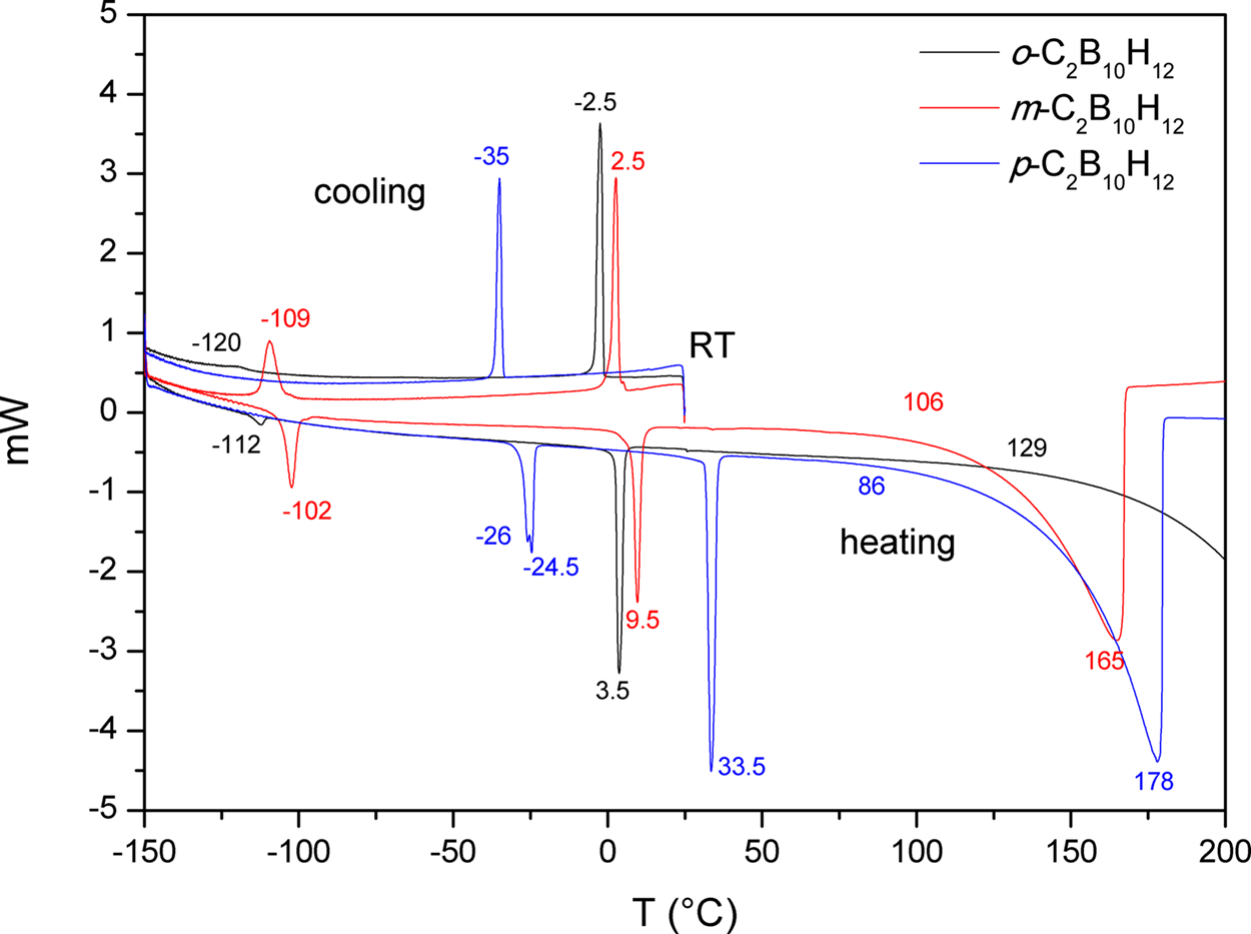
DSC data for the three isomers of C_2_B_10_H_12_. The DSC cycle starts by cooling from *rt* down to −150 °C and continues by heating up to 200 °C.

The DSC curves (Figure 2) and temperature-dependent SR-XPD (Figure S1) confirm the existence of three temperature polymorphs for each isomer. The values of transition temperatures agree within a few °C between the two experimental techniques, in good accordance also with published data (Figure 1). The differences should be attributed to the precision and systematic errors in temperature calibration for the different experimental techniques.

As the DSC measurement has been performed in an open system (nitrogen flow), sublimation was observed before melting. The onset temperature for the observable sublimation effect in DSC curves is the highest for the *o*-isomer, in agreement with its highest melting point. The temperature-dependent SR-XPD has not been extended to higher temperatures with the exception of the *m*-isomer, where the same onset of sublimation as in the DSC curves has been detected. The glass capillary used for SR-XPD can be considered as an open system as the filling of the capillary was typically below 1/3 of its volume.

The crystal structures of disordered phases I and II have been solved and refined using SR-XPD, and the structural models of the phases III as proposed from SR-XPD data were used as starting models for DFT calculations. Because for the phase III of the *m*- and *o*-isomers a significant discrepancy between the initial SR-XPD and calculated structure was found, an additional extensive search for the global energy minima was performed by DFT calculations. The procedure consisted of: (i) generation of an initial set of structures with different orientations of the molecules in the *fcc* lattice; (ii) optimization of each structure followed by symmetry analysis; and (iii) optimization of the symmetrized structures. For each polymorph, a minimum of 18 structures were generated. For the *o*-isomer, where the experimental phase III structure is a supercell of the cubic cell, an additional set of calculations was performed. Constraining the experimental unit cell shape, 64 sets of different orientations of molecules were generated, and each structure was optimized with respect to internal degrees of freedom. The symmetry was determined for each optimized structure, and these with new symmetry or modified settings were reoptimized. This procedure allows a reliable comparison with SR-XPD data and further validation of the structures. A ground-state model for phase III of the *o*-isomer and partly for the *m*-isomer has been found by this procedure, and we will use them in the following discussion. Further details of the crystal structure investigations may be obtained from the Fachinformationszentrum Karlsruhe, 76344 Eggenstein-Leopoldshafen (Germany), on quoting the depository numbers CSD-2097030-2097033, CSD-2097035-2097038 and CSD-2102455 for the DFT model of the *ortho* phase III.

The crystal symmetry of the phases I and II is the same in all three isomers (Table 1). Please, note that in the following, we will use the international notation (Hermann-Mauguin symbol), when speaking about symmetry elements of a point or space group, while the icosahedral rotation symmetry axes will be described using Schönflies notation.

**Table 1:** Symmetry, Lattice Parameters, and Unit Cell Volume (*V*) for the Three Temperature Polymorphs of the Three Solid C_2_B_10_H_12_ Isomers^a^

phase	*s.g.*	*V* (Å^3^)	*a* (Å)	*b* (Å)	*c* (Å)	β (deg)	*T* (°C)
I-*ortho*	*Fm*3̅	948.67(3)	9.82589(9)				27
I-*meta*	*Fm*3̅	955.8(1)	9.8506(4)				27
I-*para*	Fm3̅	955.769(3)	9.85033(6)				27
II-*ortho*	*Pa*3̅	908.22(4)	9.6842(2)				–11
II-*meta*	*Pa*3̅	911.91(9)	9.6973(3)				–11
II-*para*	*Pa*3̅	901.717(9)	9.66103(5)				–11
III-*ortho*	*Pc*	1720.73(2)	19.0228(3)	6.6809(5)	13.540(1)	90.238(6)	–192
III-*meta*	*C*222_1_	867.32(1)	9.6464(7)	9.5311(7)	9.4333(7)		–150
III-*para*	*P*2_1_/*c*	432.37(1)	6.7662(1)	9.3238(2)	9.4417(2)	133.457(1)	–176
*rt*-Li_2_B_12_H_12_	*Pa*3̅	877.0	9.5718(1)				29
*ht*-Li_2_B_12_H_12_	*Fm*3̅	1003.9	10.0129(1)				400

a The data of Li_2_B_12_H_12_ are given for comparison (own unpublished data).

**Figure 3:**
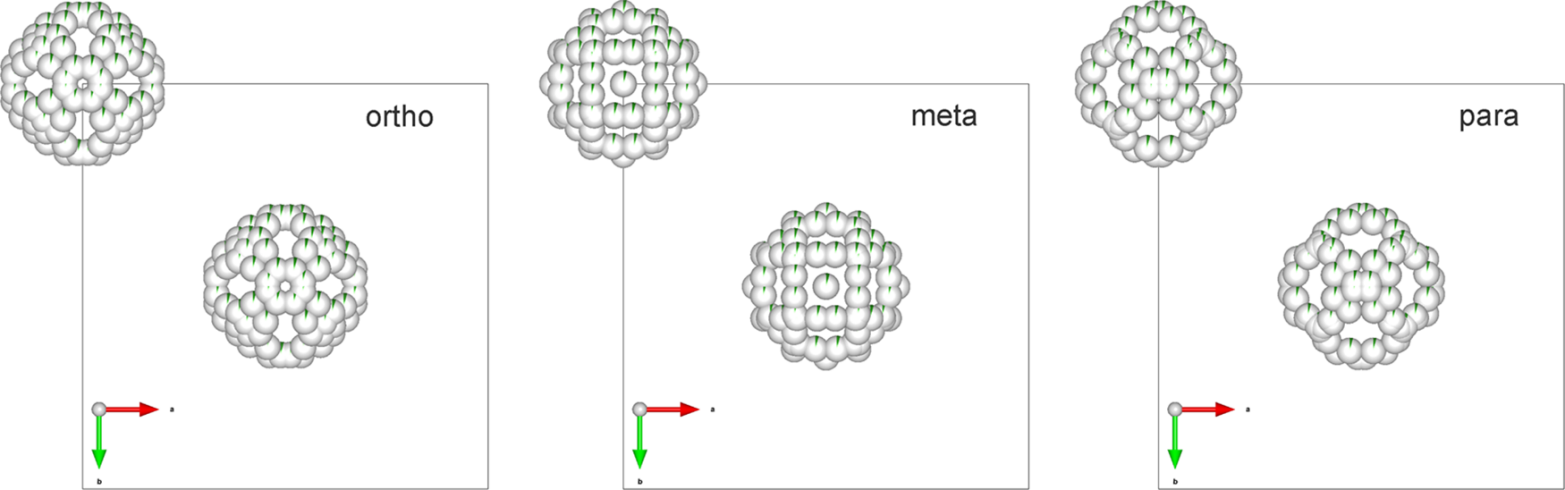
Disordered crystal structures of phase I (*Fm*3̅) for all three C_2_B_10_H_12_ isomers. Only one molecule in a unit cell vertex and one molecule in the face center are shown. Hydrogen atoms are omitted for clarity, and boron and carbon atoms are shown with the same green color.

Phase I is the cubic close packing (*ccp*) of rotating *closo*-molecules C_2_B_10_H_12_ in the cubic space group *Fm*3̅. All molecules in one structure are identical as seen by the diffraction, and orientational disorder is consistent with free isotropic rotations as proposed by NMR studies (Figure 3).

**Figure 4:**
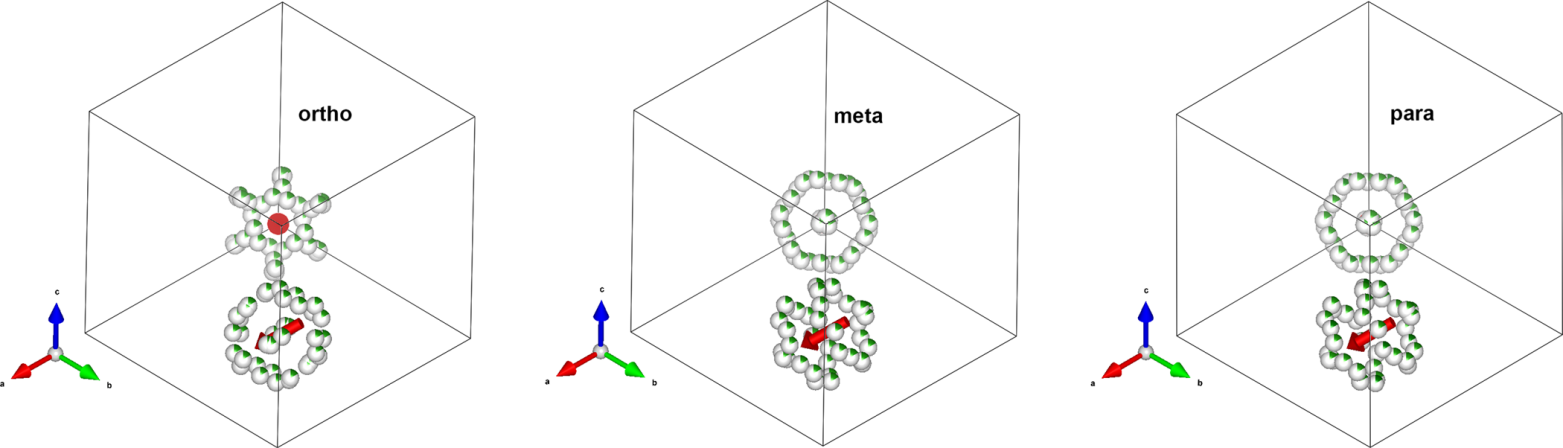
Disordered crystal structures of phase II (*Pa*3̅) for all three C_2_B_10_H_12_ isomers viewed along the crystal 3̅ rotoinversion axis *x,x,x*. Only one molecule in a unit cell vertex and one molecule in the face center are shown. Hydrogen atoms are omitted for clarity, and boron and carbon atoms are shown with the same green color. The two molecules are related by one of the *a*-glide planes. Two crystal 3̅ axes are shown by red arrows. The isomers differ in the orientation of their molecular symmetry axes with respect to the crystal 3̅ axes: While in the *o*-isomer, it is the molecular 3̅ axis, which is aligned, in the *m*- and *p*-isomers, it is the molecular 5̅ axis.

Phase II is the *ccp* of rotating *closo*-molecules C_2_B_10_H_12_ in the cubic space group *Pa*3̅. The molecules at the vertices of the unit cell are related to those in the center of faces by the *a*-glide plane, that is, the rotation axes of these two types of molecules are inclined in opposite directions *w.r.t*. the unit cell edge (Figure 4). Both molecules are rotationally disordered, revolving around one axis.

**Figure 5:**
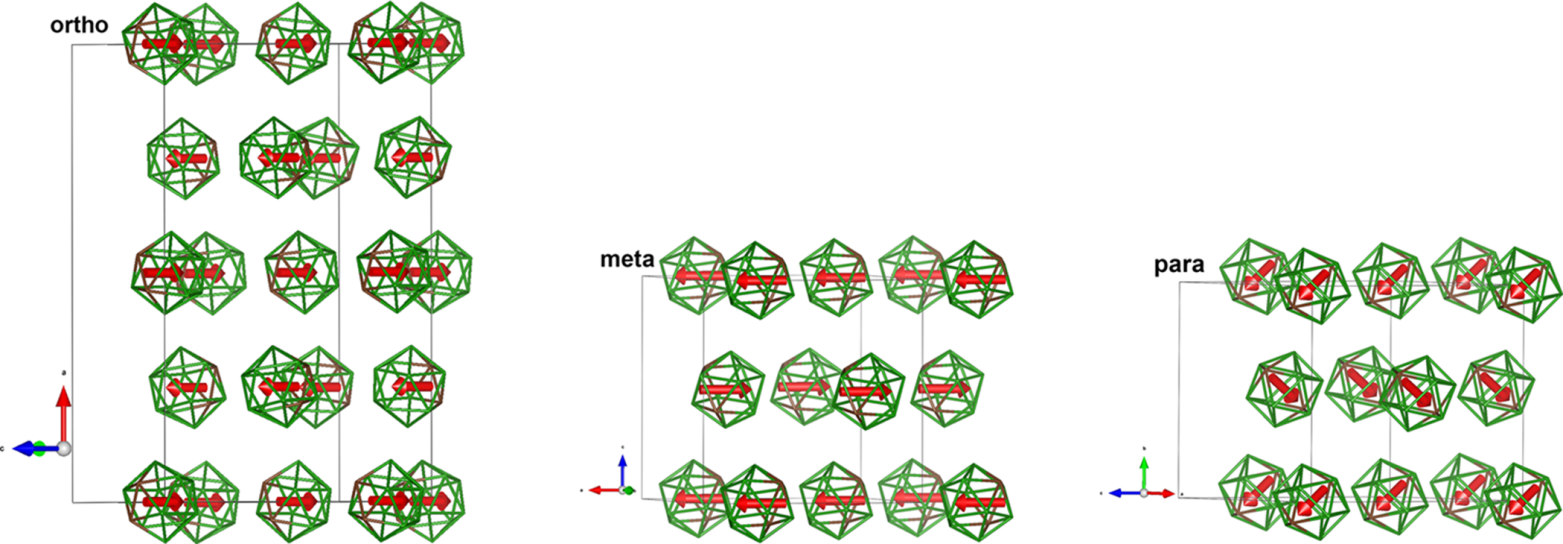
Ordered crystal structures of phase III for all three C_2_B_10_H_12_ isomers showing the fragments corresponding to the cubic subcell of the disordered phases I and II. Hydrogen atoms are omitted for clarity, and boron and carbon atoms are shown with green and brown colors, respectively. The red arrow shows the electrical dipole moment in the *o*- and *m*-isomers and C–C vector in the *p*-isomer, respectively.

Phase III is an orthorhombic (space group *C*222_1_ in *meta*) or monoclinic (space group *Pc* in *ortho* and *P*2_1_/*c* in *para*) deformation of the *ccp* unit cell, and it corresponds to an ordered crystal structure in all three isomers (Figure 5). It means that if the molecules perform rotations as suggested by NMR studies, the rotation must correspond to a jump between symmetrically equivalent positions. We have calculated energy barriers for the molecular rotations around C_2_, C_3_, and C_5_ icosahedral symmetry axes in all three isomers, which we discuss later. The ordering in the *ortho* phase III creates a twofold superstructure *w.r.t.* to the *ccp* unit cell (Figure 5) and contains four molecules in the asymmetric unit in agreement with the splitting of the ν(C–H) band in its Raman spectrum.^10^ Powder diffraction patterns obtained for the very slowly (0.1 K/min) cooled sample of the *o*-isomer did not differ from those collected with a fast rate (10 K/min). The fourth polymorph of the *o*-isomer suggested in ref (18) has not been observed.

For all three isomers, the strong exothermic peaks are visible on cooling in Figure 2 which indicates a first-order type for the transition I–II of order–disorder nature, which is related to molecule rotations. The phase transition II–III is of first order in the *m*- and *p*-isomers and of second order in the *o*-isomer as indicated by both a broad event in the DSC curve (Figure 2) and a continuous volume variation (corresponding to the continuous peak shift in the diffraction pattern, Figure S1) corresponding with the lattice parameters (Figure S2 of the Supplementary Information) for the latter. The second-order phase transition has been formulated in the *o*-isomer also by dielectric measurements.^20^ In all three isomers, the II–III transition is of an order–disorder nature accompanied by lattice deformation.

The variation of the lattice parameters and the volumes/f.u. as a function of the temperature and Rietveld plots are given in the Supporting Information (Figures S2 and S3), as well as the results calculated by DFT, which include crystal energy, cell volume, and dielectric tensors. The *p*-isomer is the most stable one in the gas phase followed by the *m*-isomer and the o-isomer, see Figure S4. The importance of dispersive interactions that bind the crystalline structures can be seen in Figure S4, where the calculated crystalline binding energy is of only −10 kJ/mol, when standard DFT gradient-corrected functionals are used, an energy being almost 10 times higher than with a proper description of the London forces.^29,30^ The size of the unit cell is largely overestimated (by >25%), once dispersion forces are neglected (Figure S4); however, even the addition of these forces overestimates the specific volume of the crystals by 9% with respect to the measured values. The origin of this discrepancy is under investigation. The dielectric properties of all three isomers are similar, see Table S1, and the dielectric constant (≈2.5) is lower than typical values for oxides or sulfides (>10).

The temperature-dependent SR-XPD of the mixture Na(CB_11_H_12_)/(*m*-C_2_B_10_H_12_) allowed identifying the presence of I- and II-type polymorphs with a transition temperature of 45 °C. The ionic conductivity increases at the transition from 10^–4^ to 5 × 10^–3^ S cm^–1^, with an activation energy in the conductive phase I of 212 meV (Figure S5). The CV measurements performed on Na_*x*_(CB_11_H_12_)_*x*_(C_2_B_10_H_12_)_1–*x*_ mixtures for composition *x* = 1/2, 1/3, and 2/3 are shown in Figure S6. Two oxidative events are visible in each sample: the former between 3 and 3.3 V and the latter peak between 4.2 and 4.4 V vs Na^+^/Na. The decomposition of NaCB_11_H_12_ can be assigned to the peak at 4.2–4.4 V, and the peak at 3.0–3.3 V has been associated with an unknown process in the NaCB_11_H_12_ pellets that nevertheless does not perturb the reversible Na^+^ shuttling throughout the solid electrolyte.^21,33^ To gain more insights into the first oxidation process, we have designed an experiment, where the impedance is measured before and after a 3.0–3.2 V scan in the CV curve. It allows understanding whether the 3.0–3.2 V peak related to an unknown process negatively affects the interfaces, that is, decreases the ionic conductivity. As shown in Figure S7, the resistance of the pellet increases from ∼0.8 kΩ measured before the peak to 4 kΩ after the peak. We conclude from our data and in agreement with literature values on the oxidative stability of the carborane^34^ that *m*-C_2_B_10_H_12_ is at least as stable as NaCB_11_H_12_.

## Discussion

The basic ideas about the thermal polymorphism in the carborane C_2_B_10_H_12_ isomers have been clear already from previous spectroscopic studies.^10−14,19,20^ With our high-resolution crystallographic studies combined with DFT calculations, we complete the full image of the bonding situation and the crystal structures of the low-temperature phase III in these exciting crystals. Starting from fully ordered phases III, the thermal motion introduces increasing rotational disorder leading to rotatory phases (plastic crystals), first in the phases II with differences between the isomers, and then in the phases I, where the rotation disorder of the molecules is isotropic in all three isomers (Figure 3). The difference between the phases II, as observed by powder diffraction, concerns the orientation of one symmetry axis of the molecular point group (boron and carbon considered as the same atoms) *I*_h_ (5̅3̅2/m) parallel to the 3̅ rotoinversion axis of the space group *Pa*3̅. In Figure 4, two 3̅ axes of the space group along the directions [111] and [11̅1̅] are shown by red arrows. While in the *o*-isomer, it is the molecular C_3_ axis that is aligned to the 3̅ axis of the space group *Pa*3̅, in the *m*- and *p*-isomers it is the molecular C_5_ axis.

The crystal structures of the ordered phases III have been determined by DFT calculations with the starting structural models determined by the powder diffraction. This allowed finding the orientation of the dipole moment in the *m*- and *o*-isomorphs and the orientation of the C–C vector (symmetry axis of the quadrupole moment) in the *p*-isomer (Figure 5). The *m*-isomer shows a simple antiferroelectric order resulting in zero macroscopic spontaneous polarization at all temperatures. The *o*-isomer shows also antiferroelectric order, similar to the *m*-isomer. Please note that this model of phase III of the *o*-isomer is one of many other models obtained by DFT optimization, albeit lowest in the energy. All these models are based on the cubic lattice deformed in a slightly different manner and creating always a twofold superstructure. The difference between the models lies in the molecule orientation. However, the energy differences between the models are very small, in the order of 0.03 eV, while kT ≈ 0.025 eV for *rt*, and a zero-point energy for molecules like C_2_B_10_H_12_ > 2 eV. We cannot exclude the coexistence of domains with different deformations of the cubic structure creating a twofold superstructure at the phase transition II-III in the *o*-isomer. Such a structural model is hardly detectable by XPD because of very low scattering contrast between boron and carbon. However, it may be related to a metastable “glassy” phase IIa of the *o*-isomer obtained by slow cooling below −108 °C and observed by Raman spectroscopy.^10^ The “glassy” phase IIa with its multiple split of the ν(CH) Raman band is in agreement with the existence of domains having different deformations of the cubic structure,^10^ while our ordered structure of the phase III containing four molecules in the asymmetric unit is in agreement with the double split ν(CH) Raman band of a monodomain antiferroelectric crystal. Verification of the existence of structural domains requires a single crystal, which is the objective of our future work. Nevertheless, formally speaking, we point out that in the phase IIa, only dipole ordering can be glassy and not the crystal structure itself because sharp Bragg peaks are clearly visible from the SR-XPD data.

**Figure 6:**
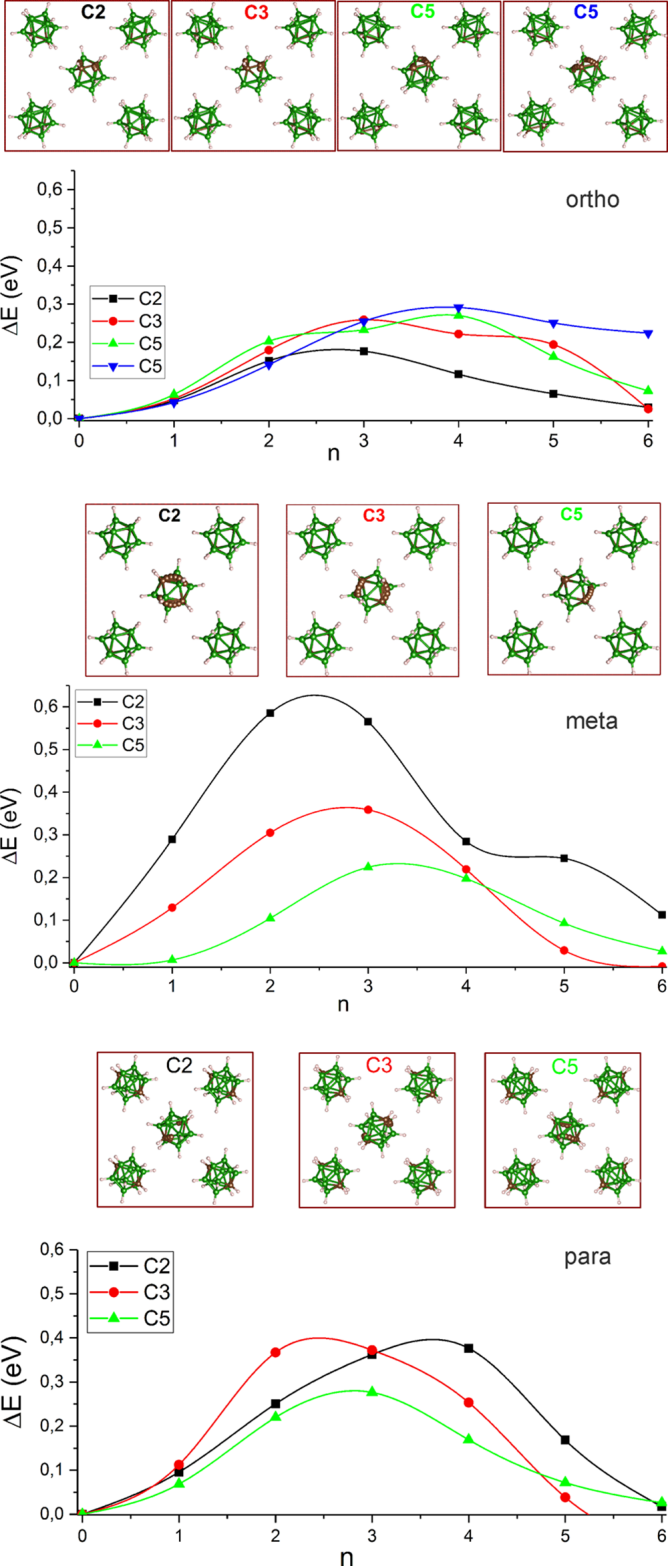
Calculated energy barriers for C_2_B_10_H_12_ rotations around the icosahedral C_2_ axis (black squares), C_3_ axis (red circles), and C_5_ axis (green, blue triangles) for the *ortho* (top), *meta* (middle), and *para* (bottom) isomers. Each rotation is visualized such that the rotating molecule is at the center of a *ccp* plane (100), and only relevant B or C atoms are visualized (boron in green, carbon in brown, and hydrogen in gray).

The calculated rotation barriers of icosahedral C_2_B_10_H_12_ molecules for the ordered phases III are presented in Figure 6. Three independent molecular axes (C_2_, C_3_, and C_5_) were considered for each isomorph, and the barriers were calculated for molecular jumps between equivalent positions of a given space group. Here, equivalent means disregarding differentiation between boron and carbon. One can notice that molecular rotations around any axis are significantly lower for the *o*-isomer (Figure 6 top). For this isomer, the rotations around the C_2_ and C_3_ axes have the lowest energy barriers of 0.18–0.25 eV. However, these rotations change the orientation of the molecule in the crystal, when the difference between boron and carbon is taken into account. The new configuration is not higher than 0.02 eV above the starting configuration of the ground state. C_5_ rotations do not change the carbon positions, if the rotation axis passes through the two carbon atoms. Once the rotation axis does not pass through carbon atoms, the final state has higher energy. For the *p*- and *m*-isomers, the rotation of C_2_B_10_H_12_ around the C_5_ molecular symmetry axis has the lowest barrier of the order ≈0.2 eV (see Figure 6 middle and bottom). The rotation trajectories are shown for all considered rotations in Figure 6, where they are presented such that one (100) plane of the *ccp* molecular packing in phase I is shown irrespective of the ground-state symmetry of the isomer. For the *p*- and *m*-isomers, one of the C_5_ molecular axis coincides with the cubic space diagonal [111] in agreement with diffraction results, and this axis is preferred for molecular jumps. For the *o*-isomer, the C_3_ molecular symmetry axis is aligned with the cubic space diagonal [111] as also observed by powder diffraction, and the molecules are aligned in such a way that the dipole moment with C_2_ molecular symmetry is aligned along the *ccp* [011] direction. This leads to more complex rotation patterns for the *o*-isomer, while rotations around the C_5_ axis dominate in the other isomers.

Our results do not exclude isotropic rotations in the *p*-isomer as claimed from NMR studies, but C_5_ rotations seem to be preferred, which is in agreement with ref (19). In contrast, a disagreement between NMR and our results appears for the *o*-isomer, where NMR studies show predominantly a rotation around C_2_ axis with decreasing temperature, while our results show C_3_ axis rotations. As the 3̅ axis of the space group and molecular C_3_ axis nearly coincide in the *o*-isomer (Figure 3), this is the only symmetry element presented on the Wyckoff site 4*a* of space group *Pa*3̅, where the molecule is located, phase II of the *o*-isomer is nearly ordered. A reorientation model has been formulated and verified by NMR for the *o*-isomer in ref (14). It is based on a small deviation (few degrees) of the molecular C_3_ axis from the 3̅ axis of this space group. As this misorientation vanishes with decreasing temperature, which would lead to a perfect order in the *Pa*3̅ group as observed for example in Li_2_B_12_H_12_ (ref (35) and references therein), another interaction has to become dominating at lower temperatures. Dipole–dipole interaction might be at the origin of continuous decrease of C_3_ and C_5_ rotations and lattice deformation, driving the second-order phase transition into the phase III. In fact, the crystal structure of phase III of the *o*-isomer determined here reveals ordering of the molecular dipole moment along the [011] *ccp* direction in such a way that certain C_3_ and C_2_ molecular axes are approximately aligned with the 3̅ axes of the *ccp* unit cell. For this isomer, only C_2_ rotations, which do not reorient the dipole moment, are allowed in the phase III and lead to the lowering of the symmetry to monoclinic and creation of the twofold superstructure. Why a similar mechanism of the second-order phase transition does not operate in the *m*- and *p*-isomorphs is not clear. One explanation could be the biggest dipole moment in the *o*-isomorph, and the other is simply based on the difference in charge distribution, that is, carbon positions, in the icosahedral molecule. The very low barriers for reorientation of the *o*-isomer C_2_B_10_H_12_ molecules without clear preference of rotation direction can be also a driving force for a continuous phase transition of the second order. The phase III of the *p*-isomer without the dipoles is then an example of pure charge distribution interaction.

Polymorphism in the three C_2_B_10_H_12_ isomers serves as a case study of two interactions in compounds containing icosahedral boron-hydrogen *closo*-anions. It was proposed that two effective interactions are crucial for ionic conductivity in this class of materials:^36^ (i) isotropic cohesive interaction (of dispersive origin in molecular C_2_B_10_H_12_, with additional Coulombic component in metal salts) and (ii) anisotropic local interaction, which results from nonuniform charge distribution around icosahedral clusters. The local interactions may be averaged out by thermally activated cluster dynamics (rotations and jumps), which effectively increases the symmetry of the cluster.

We may compare our results with metal hydridoborates and their carbon-derivatives containing B_12_H_12_^2–^ and CB_11_H_12_^–^ anions, respectively. Two thermal polymorphs with the same space group symmetry as I and II occur in Li_2_B_12_H_12_ (ref (35) and references therein) (Table 1). Similarly, to all *ht*-C_2_B_10_H_12_ isomers, these anions are orientationally disordered in phase I equivalent (*Fm*3̅*m*), and cations do not have definitive positions in the lattice; they are confined to migrate between tetrahedral voids. The orientational disorder in *ht*-Li_2_B_12_H_12_ may be modeled as a rotation around C_3_ by 45°, which is not a symmetry operation of the space group *Fm*3̅*m*.^35^ As the reorientation dynamics in Li_2_B_12_H_12_ has not yet been studied, we cannot conclude, whether the disorder is of dynamic or static nature, although the former is more likely. Upon cooling, at 355 °C Li_2_B_12_H_12_ undergoes a phase transition to *Pa*3̅ (phase II equivalent), where the anions are locked in the orientation having mutual 45° inclination with respect to the C_3_ axis, resulting from the *a*-glide plane. The Li^+^ cations are confined at the tetrahedral facets, where they are separated from the nearest hydrogen atoms by 2.07 and 2.21 Å, such that skewed octahedral coordination is present around each anion (Figure S8). This atomic distribution is pinning anions and rotations are subject to large barriers. Because no lower symmetry polymorph has been reported for Li_2_B_12_H_12_ at lower temperatures,^35^ it can be argued that what drives the I-II type transition in both C_2_B_10_H_12_ and Li_2_B_12_H_12_ is the local perturbation of the isotropic cohesive interaction in phase I.

Because formally, B_12_H_12_^2–^ has no net electrical dipole or higher moment, this perturbation in Li_2_B_12_H_12_ arises from the proximity of Li^+^ cations. Thus, the difference between Li_2_B_12_H_12_ and C_2_B_10_H_12_ is canceled out in phase I, while moving to phase II, the former results in a fully ordered structure, while the latter performs uniaxial rotation, with strong preference for C_5_ reorientation in the *p*- and *m*-isomers. This structural behavior can be directly compared to the order–disorder phase transition in crystalline C_60_, which shows partial rotational ordering below −13 °C by lowering the symmetry from *Fm*3̅ (phase I) to *Pa*3̅ (phase II) with the molecules locked in two nonequivalent orientations. The origin of this optimal arrangement of C_60_ molecules is explained by anticlockwise molecule rotation by ≈98° around the [111] direction. This minimizes intermolecular electrostatic interactions between molecules in a way that pentagon faces hexagon–hexagon bonds of the nearest molecule.^37^ Such a small charge perturbation is sufficient for freezing some rotational degrees of freedom in C_60_. Similarly, in phase II of C_60_, the uniaxial jumps between symmetrically equivalent orientations around the C_3_ axis are observed by NMR. The fraction of one nonequivalent orientation is decreasing with decreasing temperature below −183 °C (second-order phase transition into phase III). A glassy state with two frozen orientations may occur.^37^

**Figure 7:**
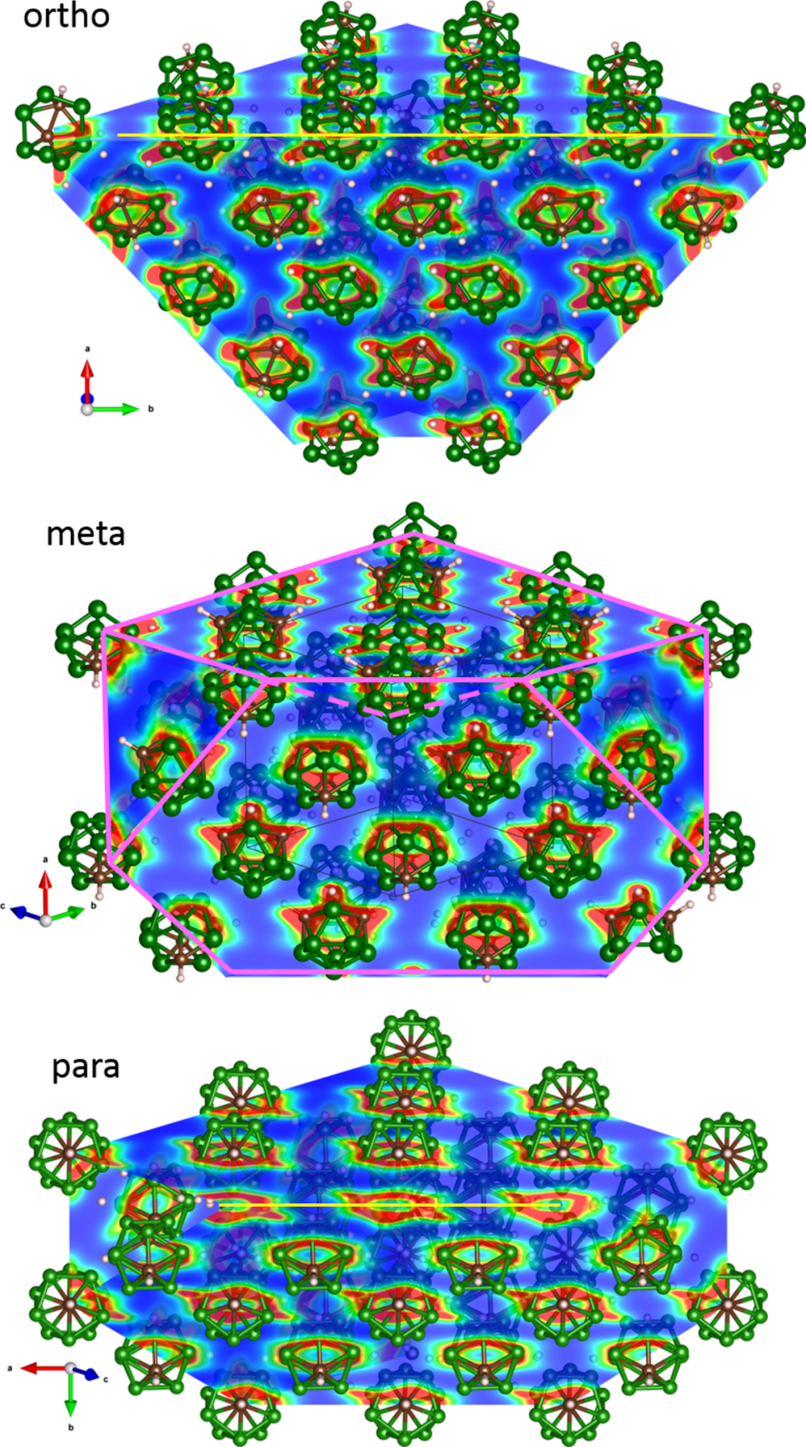
Cross section of charge density for the ground-state structures of the *ortho*- (top), the *meta*- (middle), and the *para*-isomer (bottom) of C_2_B_10_H_12_. The charge density is plotted from 10^–4^ e/Å^3^ (blue) to 10^–1^ e/Å^3^ (red); the cross section is presented with respect to the *fcc* lattice as shown in the middle plot with magenta lines.

The situation changes, when the anion cluster carries a dipole or higher electrical moment thus it is effectively asymmetric: It can be seen comparing LiCB_11_H_12_ and Li_2_B_12_H_12_. Indeed both have the same disorder structure (*Fm*3̅*m*) at high temperature. LiCB_11_H_12_ in contrast, below the transition temperature, shows an ordered structure that is orthorhombic (*Pca*2_1_) deformation of *ccp.* In this case, the phase II equivalent is skipped, and the perturbation of isotropic interaction by the less symmetrical distribution of Li^+^ cations (number of lithium cations in the crystal is reduced by half) leads directly to the phase III equivalent. The local coordination of CB_11_H_12_^–^ is shown in Figure S8 with three Li^+^ coordinating the anion. They are separated from the nearest hydrogen atoms by 2.01 and 2.37 Å, even at a shorter distance than in Li_2_B_12_H_12_. The strong electrostatic interaction and asymmetry of ion distribution lead to an orthorhombic deformation of the *fcc* lattice. This asymmetry suggests that the II–III transition in C_2_B_10_H_12_ is induced by dipole–dipole or quadrupole–quadrupole interaction as suggested in ref (13). or simply by a strongly inhomogeneous charge distribution presented in Figure 7, which reveals further differences between isomers. While all molecules are icosahedral, the distribution of carbon atoms brings the 5̅ dominant symmetry for the *p*-isomer, 2/**m** for the *o*-isomer and a somehow more complex symmetry pattern for the *m*-isomer. Because this charge distribution asymmetry is much larger in C_2_B_10_H_12_ than for C_60_, the driving force for molecular ordering is stronger.

While the thermal polymorphism of Li_2_B_12_H_12_ and LiCB_11_H_12_ is quite similar to that for C_2_B_10_H_12_ because of the small ionic radius of Li^+^ (0.59 Å), the situation changes for salts containing larger cations such as Na^+^ (0.99 Å, ionic radii according to ref (38).). For Na_2_B_12_H_12_, the distortion of the anion coordination octahedra is large, it cannot match small deformations of the *fcc* lattice, and a monoclinic deformation is observed (Figure S8). The ordered *rt* phases in sodium compounds are a deformation of the *ccp* anion sublattice, but while Na_2_B_12_H_12_ is monoclinic (*P*2_1_/*c*), NaCB_11_H_12_ crystallizes isostructurally to LiCB_11_H_12_, that is, ordered orthorhombic (*Pca*2_1_) deformation of *ccp*. For these phases, the asymmetric distribution of three cations around a nonspherical charge distribution of CB_11_H_12_^–^ stabilizes the lattice deformation and locks the anion rotation (Figure S8) already at *rt*. Phase II is skipped in both systems, and the phase I equivalent appears above 107 °C for NaCB_11_H_12_ and above 256 °C for Na_2_B_12_H_12_ as a metastable phase in the latter (ref (34) and references therein).

In A_2_B_12_H_12_ salts with even bigger alkali-metal cations (A = K, Rb, Cs), the ordered phase III equivalents are also stabilized at *rt*, but as a true anion *ccp* with *Fm*3̅ symmetry. For these heavier alkali-metals, the *closo*-hydroborate anions are surrounded by eight cations and no distortion of the *fcc* lattice is present (Figure S8). Each cation is equidistant to three nearest hydrogen atoms; thus the C_3_ molecular axis is aligned with the 3̅ axis of the cubic unit cell. Please note that the Wyckoff site 4*a* symmetry (*m*3̅), where the anion is located is compatible with the icosahedral symmetry, allowing an ordered orientation of the anion. This is possible because the angle between each of the three normal vectors to the three mirror planes and 3̅ axis is of 54.74°, as it is in the regular icosahedron. For such an orientation, the icosahedral C_2_ axes are aligned along principal axes of the cubic unit cell. Increasing the symmetry to *Fm*3̅*m*, the site symmetry *m*3̅*m* is no longer compatible with the icosahedral one, thus resulting in a disorder orientation, as for the case of *ht*-Cs_2_B_12_H_12_.

## Conclusions

The crystal structures of the three thermal polymorphs, I (high-temperature), II (middle-temperature), and III (low-temperature), existing for each isomer of C_2_B_10_H_12_ (1,2-*ortho*, 1,7-*meta,* and 1,12-*para*) have been determined by X-ray powder diffraction and DFT calculations. The crystalline structure of these materials is bound by weak dispersive interactions between *closo*-dicarbadodecaborane molecules. The crystal structures are in agreement with previous calorimetric, NMR, IR, Raman, and dielectric spectroscopic studies. They are also directly comparable to crystalline C_60_ as both molecules have the same icosahedral symmetry. The difference between rotatory phases I and II is in isotropic rotations of C_2_B_10_H_12_ in the former and anisotropic rotations in the latter. The preferred rotational axis in II varies with the isomer, and it is C_5_ for the *meta* and the *para* and C_3_ for the *ortho*-isomer. The ordered phases III are orthorhombic (*meta*) or monoclinic (*ortho* and *para*) deformations of the cubic unit cell of the disordered phases I and II. The ordering in the *ortho*-phase III creates a twofold superstructure *w.r.t.* the cubic unit cell of the disordered phases I and II. The ordering scheme is from the molecular shape point of view (not regarding the carbon positions) identical for all three isomers and similar to the Li_2_B_12_H_12_ case. *Meta* and *ortho* isomers become disordered at similar temperatures, while *para*-C_2_B_10_H_12_ needs further thermal energy to disorder. The metastable phase IIa observed in ref (10) for the *o*-isomer, which shows multiple split bands in the Raman spectrum, was not confirmed in this work. It is suggested that it corresponds to a multidomain crystal with domains having different dipole ordering schemes and different deformations of the cubic unit cell for the disordered phases I and II leading always to a twofold superstructure. The energy landscape for molecular misorientation is very shallow (≈ 0.01 eV/atom) for this isomer. Such a crystal would have an X-ray powder diffraction pattern that is not possible to distinguish from that of a monodomain crystal of phase III.

The intercluster interactions in compounds made from icosahedral boron-hydrogen *closo*-clusters have been divided into two categories allowing the explanation of thermal polymorphism in the C_2_B_10_H_12_ carborane and related metal salts: (i) dispersive isotropic cohesive interaction of molecular C_2_B_10_H_12_, with an additional Coulombic component in metal salts, and (ii) anisotropic local interaction resulting from nonuniform charge distribution around icosahedral clusters. The local interactions may be averaged out by thermally activated cluster dynamics (rotations and orientation jumps), which effectively increases the symmetry of the cluster. The contribution of local interactions (cation–anion attraction) to the ordering in crystals is stronger in alkali-metal salts containing icosahedral boron-hydrogen *closo*-clusters as compared to C_2_B_10_H_12_. This results in fully ordered structures at *rt*. With the exception of Na^+^ (monoclinic deformation), the ordered structures of alkali-metal *closo*-dodecahydridoborates are cubic with anions packed in *ccp*. Anion clusters carrying a dipole moment and anisotropic charge distribution such as CB_11_H_12_^–^ lead to a deformation of the cubic symmetry.

The carborane C_2_B_10_H_12_ resists at least as strongly as the CB_11_H_12_^–^ anion to the oxidation, and both clusters form easily mixed compounds. This allows designing solid electrolytes such as Na_*x*_(CB_11_H_12_)_*x*_(C_2_B_10_H_12_)_1**–***x*_ where the cation content may be varied.

## Supporting Information Available

Temperature-dependent X-ray powder diffraction patterns, Rietveld plots, lattice parameters as a function of temperature, DFT calculated energy and electrical dipole moment, electrochemical data, and anion coordination in selected closo-hydridoborates (PDF)

## Accession Codes

CCDC 2097030–2097038 and 2102455 contain the supplementary crystallographic data for this paper. These data can be obtained free of charge via www.ccdc.cam.ac.uk/data_request/cif, or by emailing data_request@ccdc.cam.ac.uk, or by contacting The Cambridge Crystallographic Data Centre, 12 Union Road, Cambridge CB2 1EZ, UK; fax: +44 1223 336033.
