# Supplementary material for: Structural Phase Transitions in closo-Dicarbadodecaboranes C2B10H12
Source: Inorg Chem. 2022 Apr 1;61(15):5813–23. doi: 10.1021/acs.inorgchem.1c04022 (PMC9019807; doi:10.1021/acs.inorgchem.1c04022)
Supplement: Supplementary file 1 — ic1c04022_si_002.pdf [file ic1c04022_si_002.pdf]

Supporting Information

Structural phase transitions in *closo*-dicarbadodecaborane $C_2B_{10}H_{12}$

[a]Matteo Brighi, [a]Fabrizio Murgia, [b]Zbigniew Łodziana* and [a]Radovan Černý*

[a]Department of Quantum Matter Physics, Laboratory of Crystallography, University of Geneva, Quai Ernest-Ansermet 24, CH-1211 Geneva, Switzerland

[b]Polish Academy of Sciences, Institute of Nuclear Physics, ul. Radzikowskiego 152, 31-342 Krakow, Poland

*Zbigniew Łodziana, zbigniew.lodziana@ifj.edu.pl

*Radovan Černý, Radovan.Cerny@unige.ch

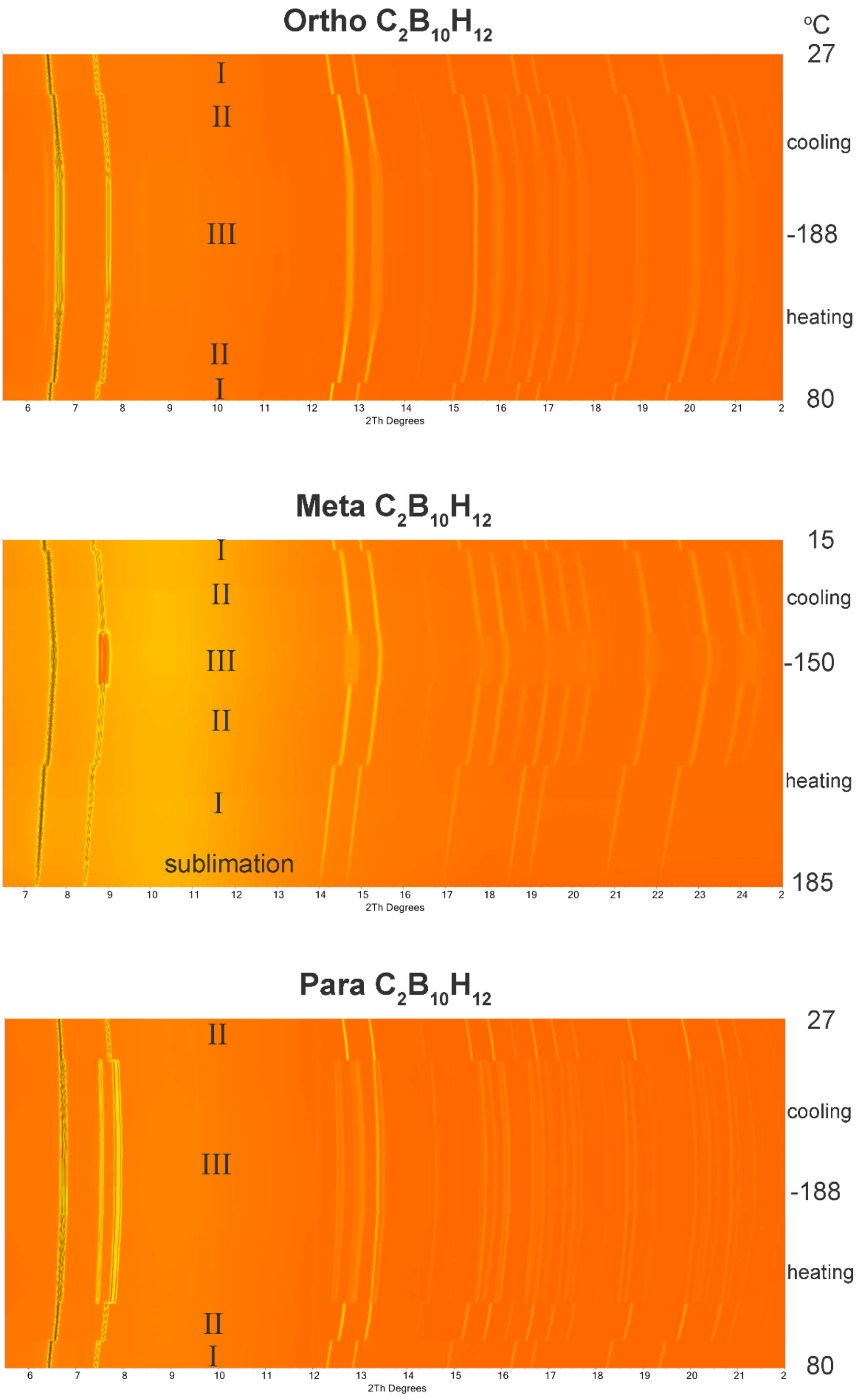

Figure S1: SR-XPD data as a function of temperature for the three isomers of $C_2B_{10}H_{12}$ (SNBL, ESRF Grenoble). X-rays wavelength λ = 0.64113 Å (*ortho* and *para*) and λ = 0.7399 Å (*meta*).

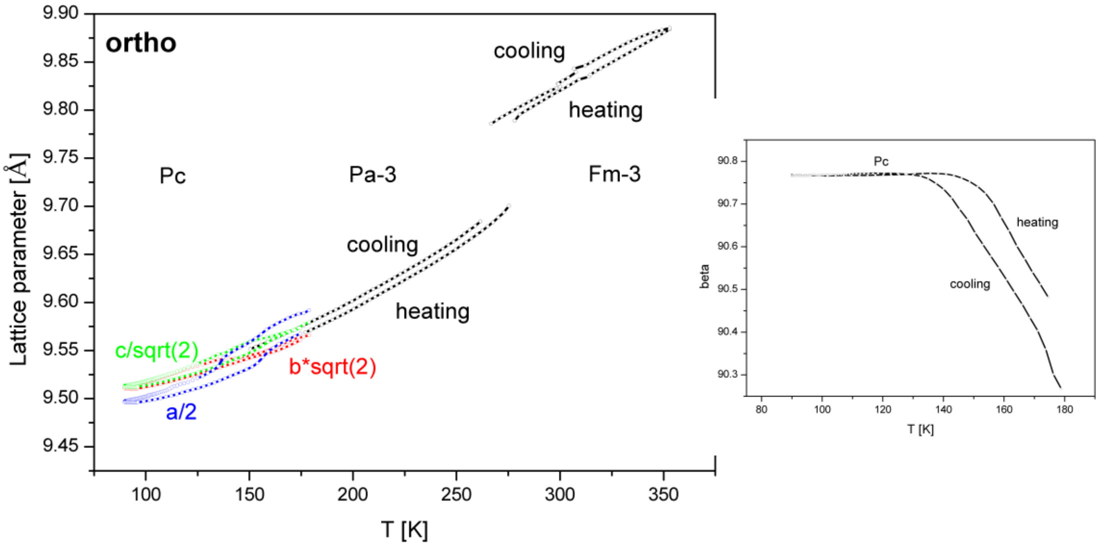
ortho
cooling
heating
Pc
Pa-3
Fm-3
cooling
heating
c/sqrt(2)
b*sqrt(2)
a/2
Lattice parameter [Å]
T [K]
Pc
heating
cooling
beta
T [K]

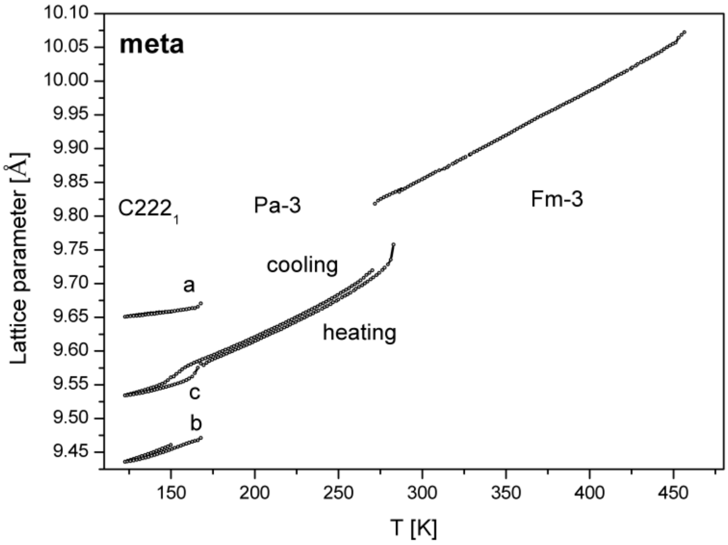
meta
C222$_1$
Pa-3
Fm-3
cooling
heating
a
c
b
Lattice parameter [Å]
T [K]

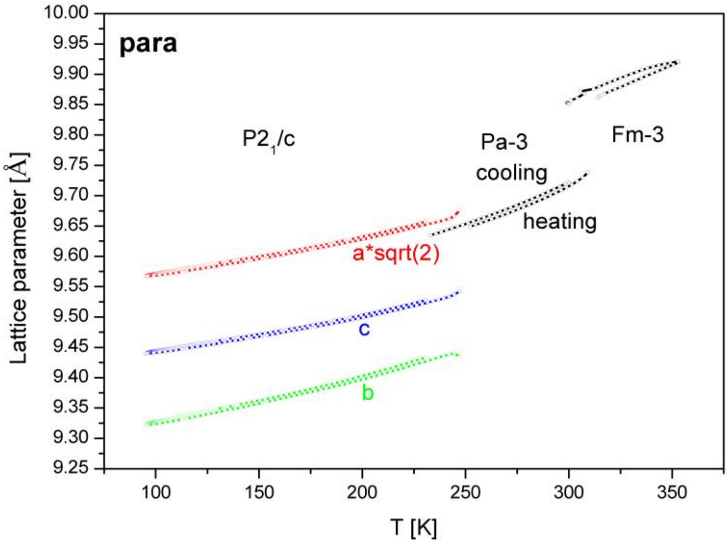
para
P2$_1$/c
Pa-3
cooling
heating
Fm-3
a*sqrt(2)
c
b
Lattice parameter [Å]
T [K]

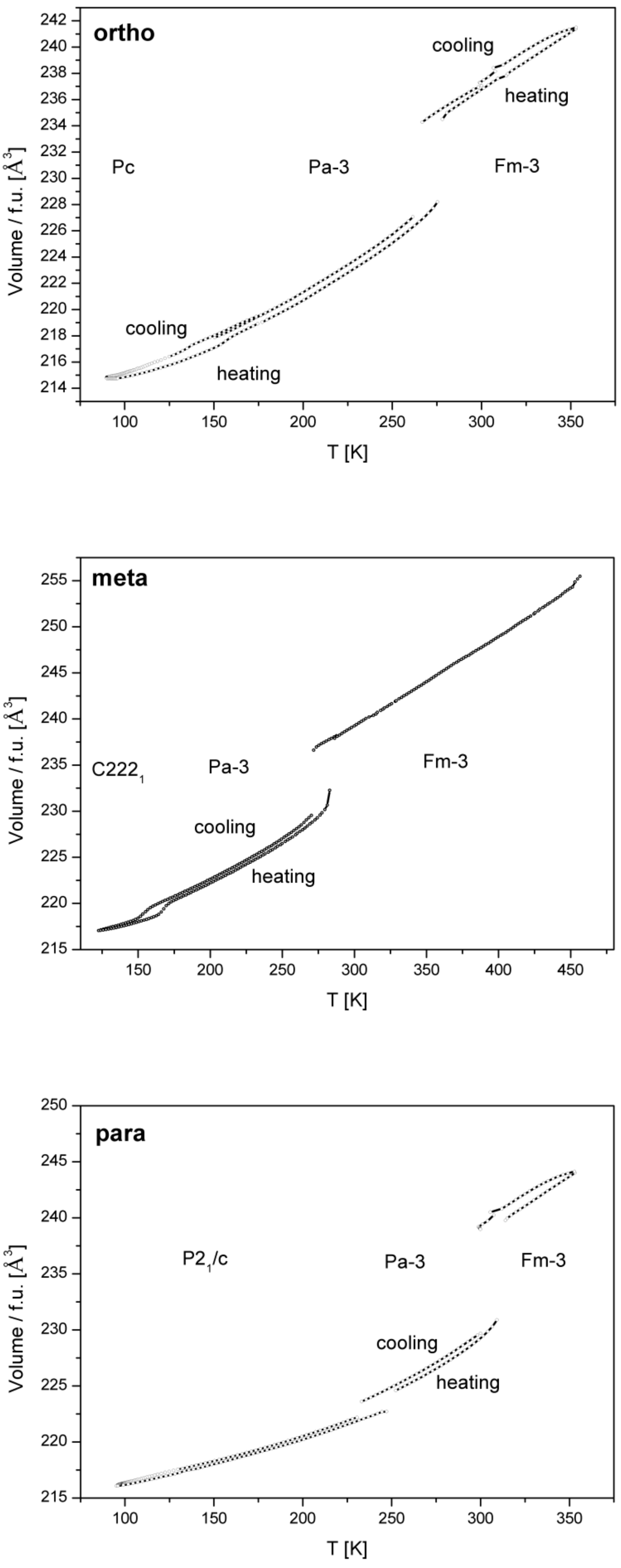

Figure S2: Lattice parameters and volume / formula unit as a function of temperature for the three $C_2B_{10}H_{12}$ isomers.

Ortho-I

Data from SNBL, T= 27 deg C, λ=0.64113 Å, R_{wp}(corrected for background)= 7.7 %, χ^2= 15.

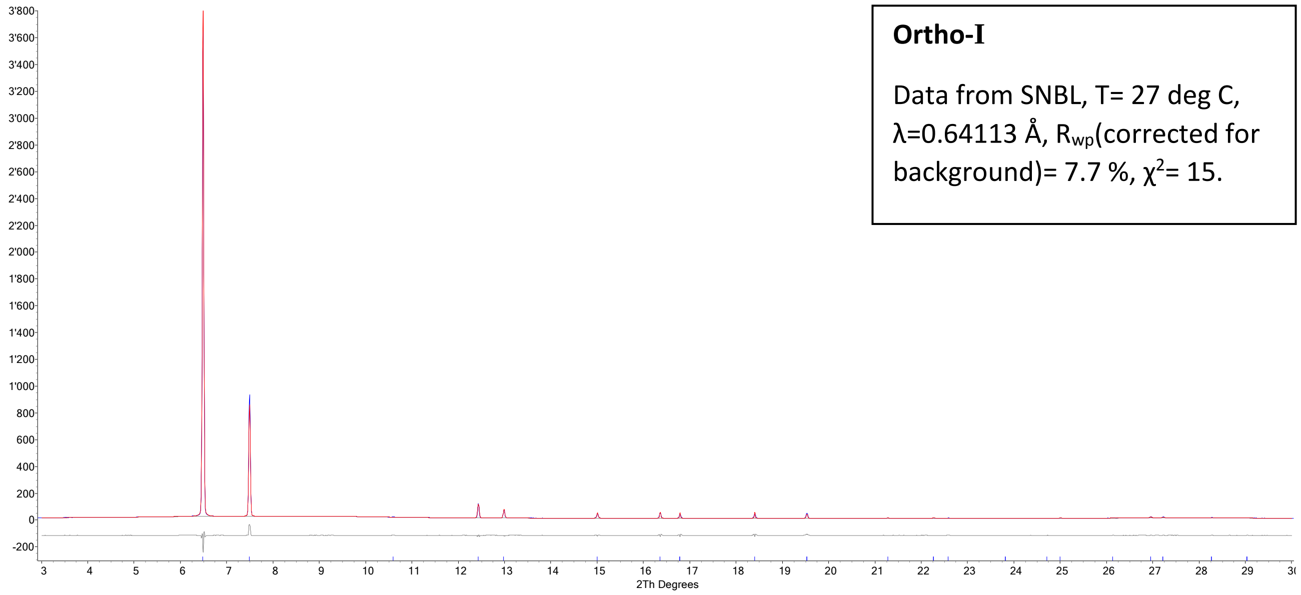

Meta-I

Data from SNBL, T= 27 deg C, λ=0.7399 Å, R_{wp}(corrected for background)= 7.7 %, χ^2= 60.

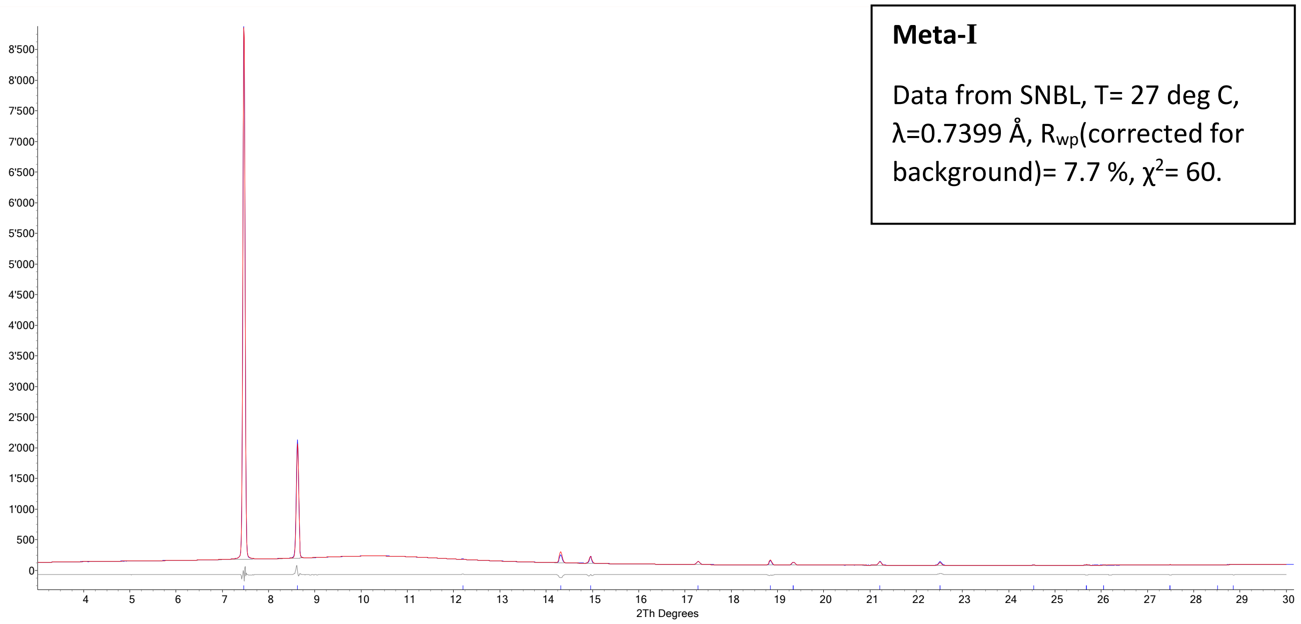

Para-I

Data from SNBL, T= 27 deg C, λ=0.64113 Å, R_{wp}(corrected for background)= 7.6 %, χ^2= 12.

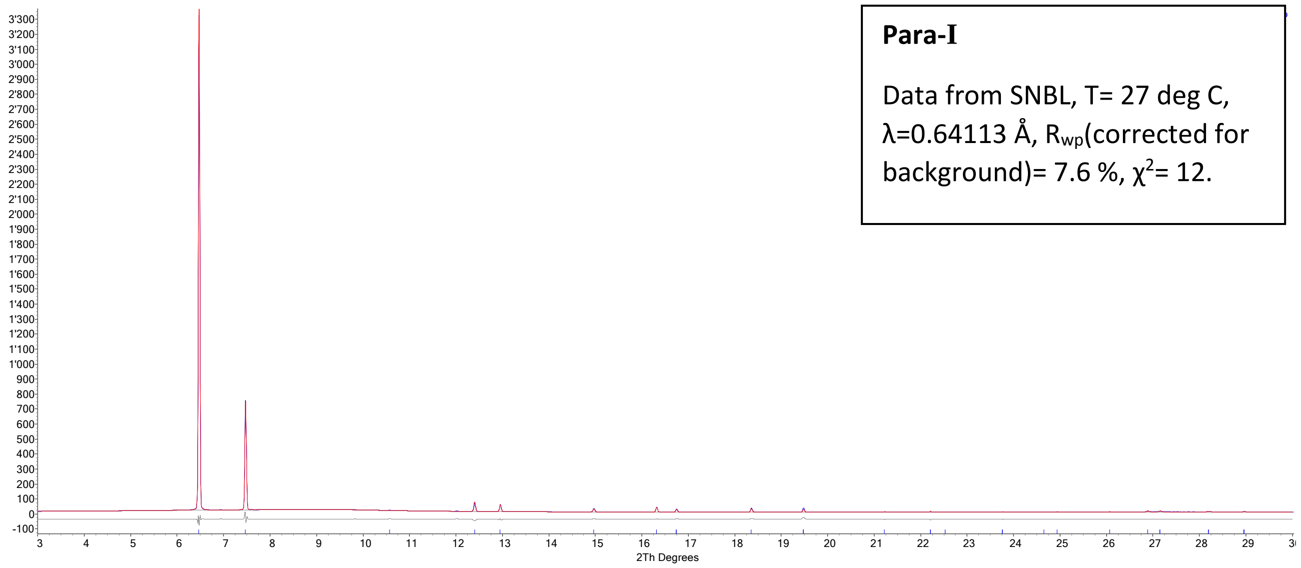

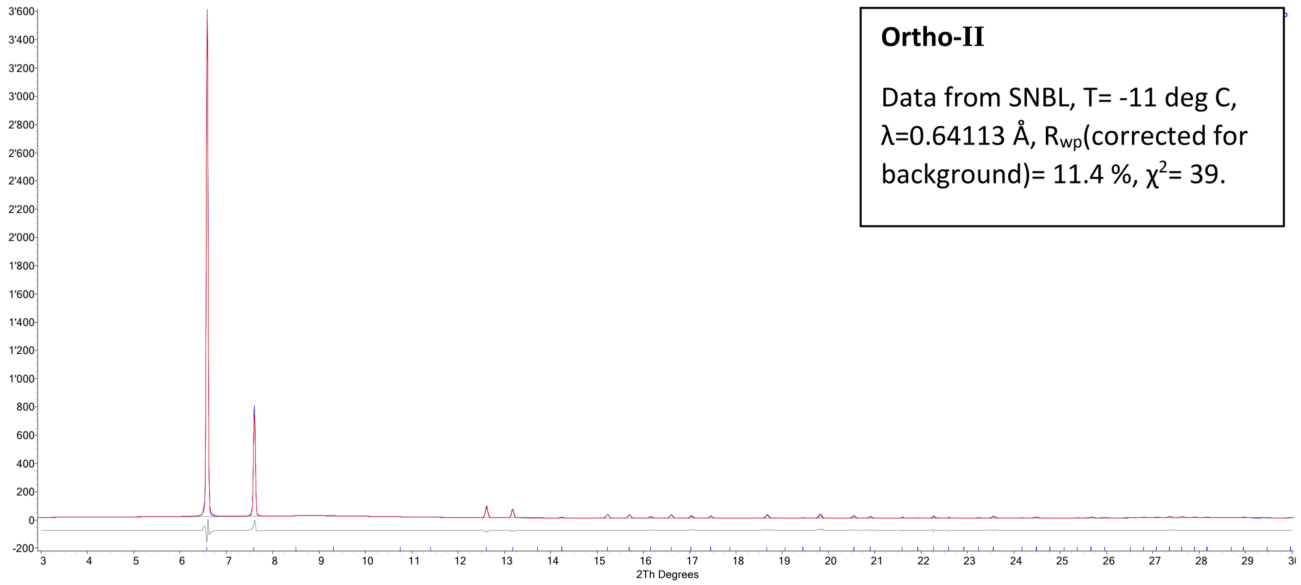
Ortho-II
Data from SNBL, T= -11 deg C, λ=0.64113 Å, R_{wp}(corrected for background)= 11.4 %, χ^2= 39.
2Th Degrees

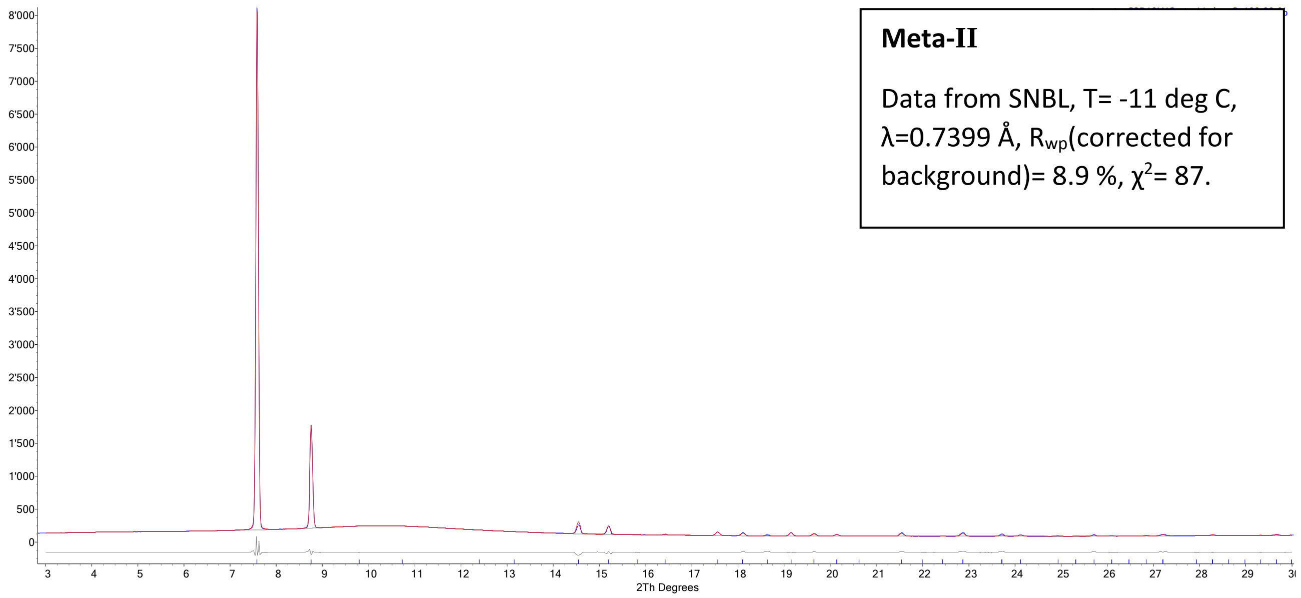
Meta-II
Data from SNBL, T= -11 deg C, λ=0.7399 Å, R_{wp}(corrected for background)= 8.9 %, χ^2= 87.
2Th Degrees

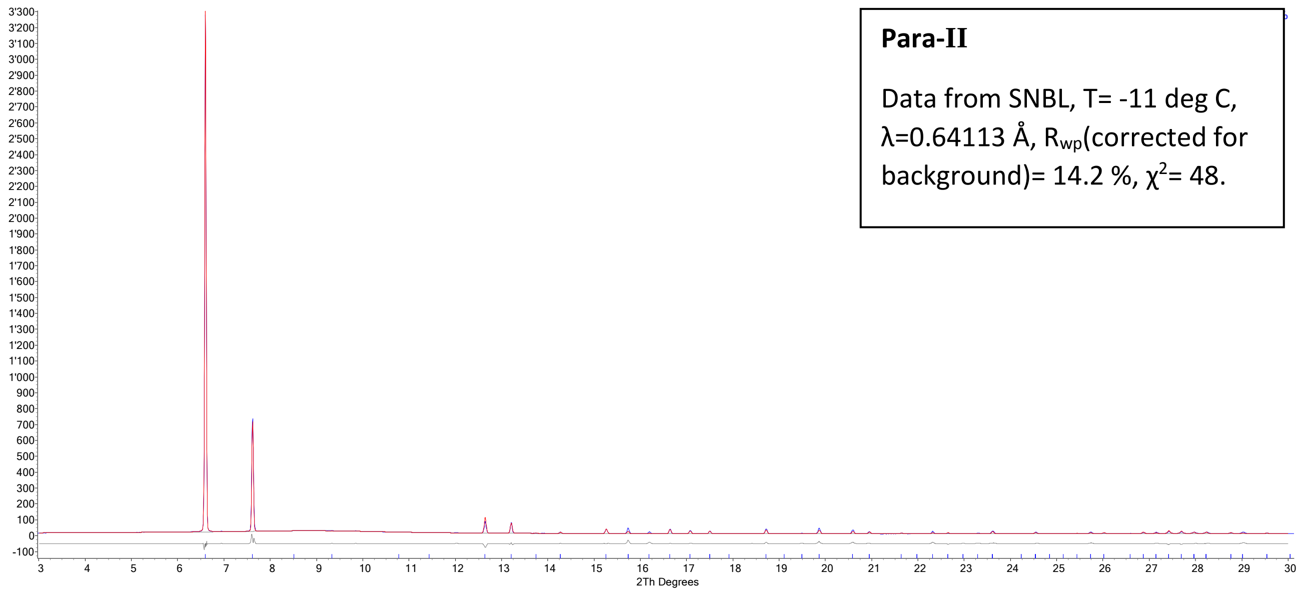
Para-II
Data from SNBL, T= -11 deg C, λ=0.64113 Å, R_{wp}(corrected for background)= 14.2 %, χ^2= 48.
2Th Degrees

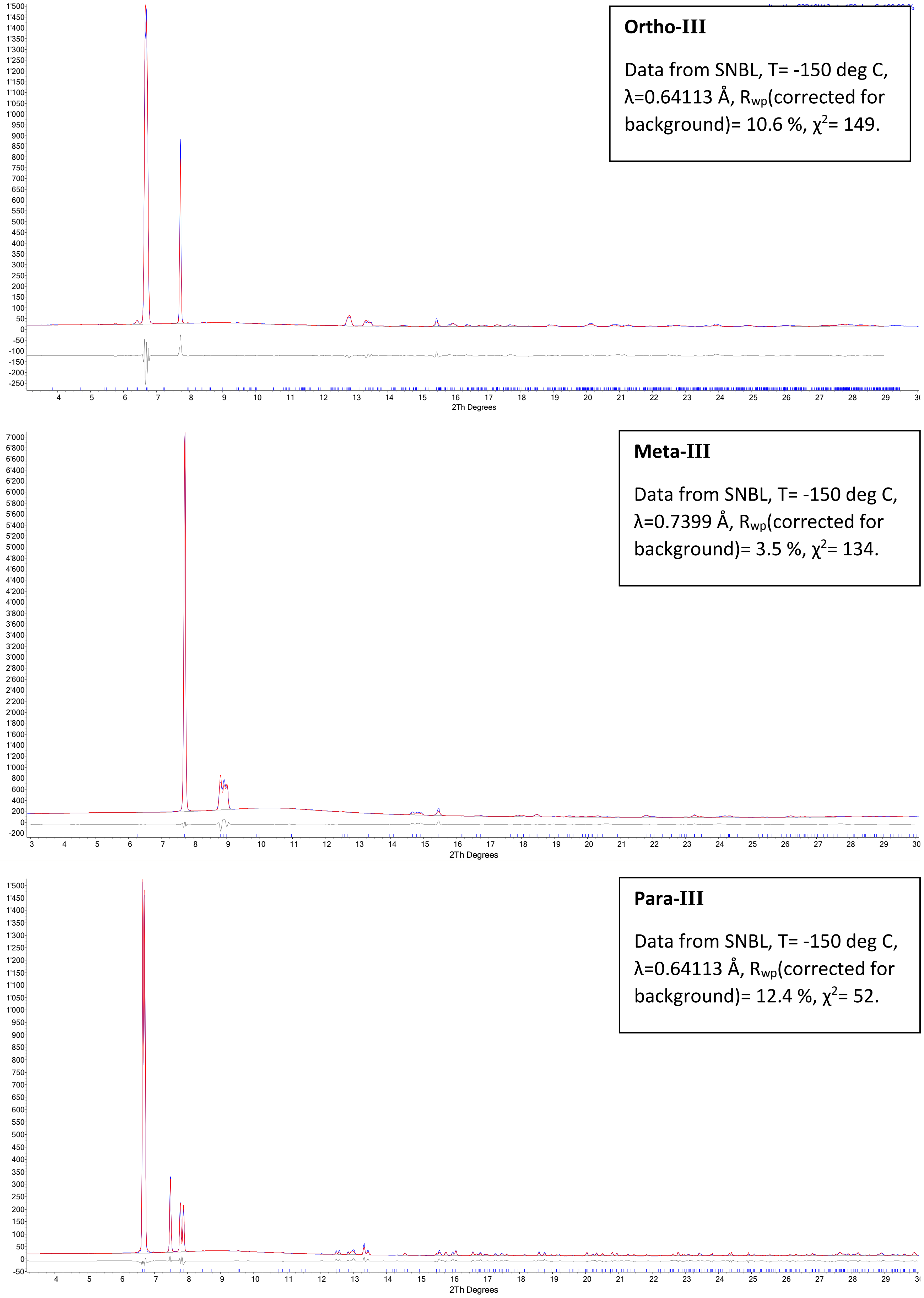

Figure S3: Rietveld plots.

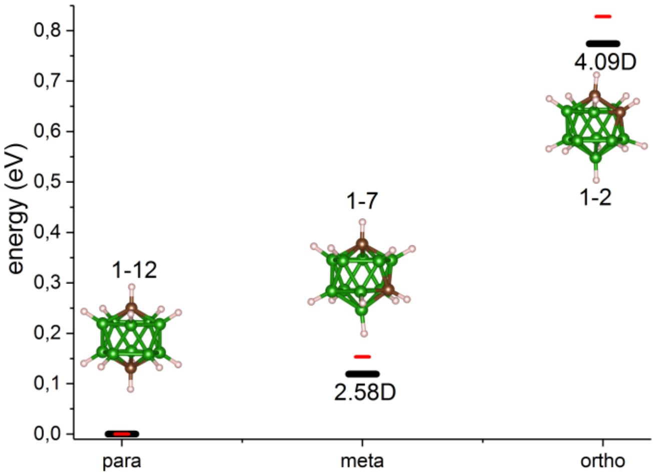

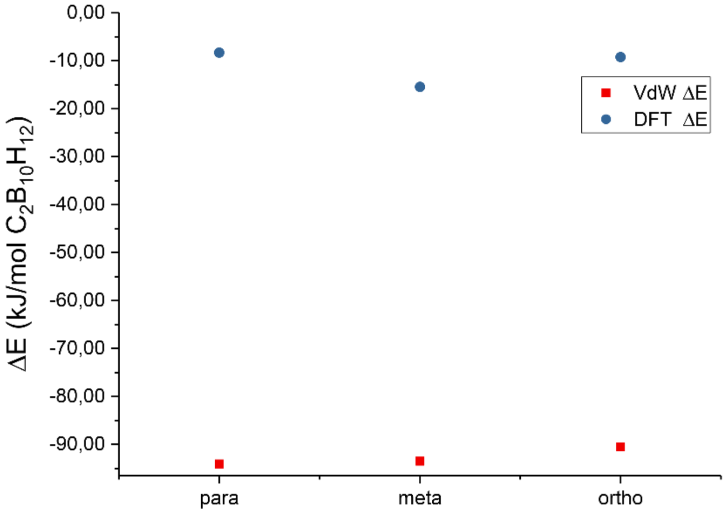

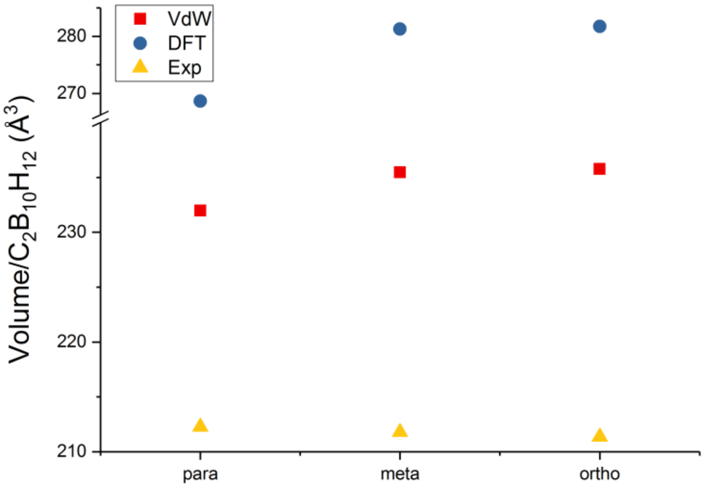

Figure S4: Top: Calculated energy and dipole moments for isolated isomers, the thin red bars show the stability of isomers as calculated in [1]; Middle: the cohesive energy of the crystal calculated with and without dispersive interactions with reference to molecules; Bottom: comparison of the volume / f.u. in three $C_2B_{10}H_{12}$ isomers from experimental data (yellow triangles), calculations including dispersive interactions (red squares), and standard DFT calculations (blue circles), note the vertical axis break.

PARA

2.560	0.000	0.002
0.000	2.536	0.000
0.002	0.000	2.494

ORTHO

2.505871	0.000000	-0.017164
0.000000	2.499403	0.000002
-0.017164	0.000002	2.517730

META

2.503	0.000	0.000
0.000	2.511	0.000
0.000	0.000	2.502

Table S1: Calculated dielectric tensors for three $C_2B_{10}H_{12}$ isomers.

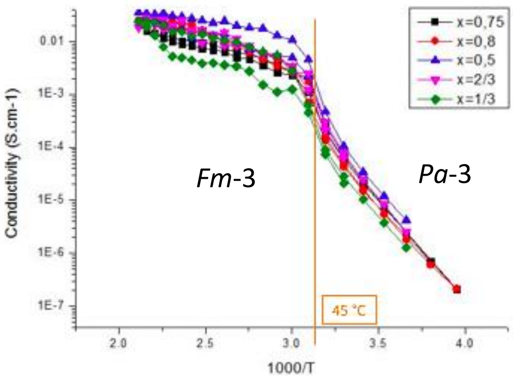

Figure S5: Ionic conductivity in mixtures $Na_x(CB_{11}H_{12})_x(m\text{-}C_2B_{10}H_{12})_{1-x}$.

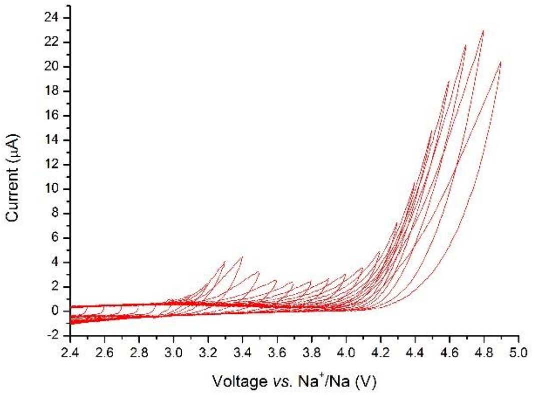

Figure S6: Cyclic voltammetry of mixture $Na_{0.33}(CB_{11}H_{12})_{0.33}$($m$-$C_2B_{10}H_{12})_{0.66}$.

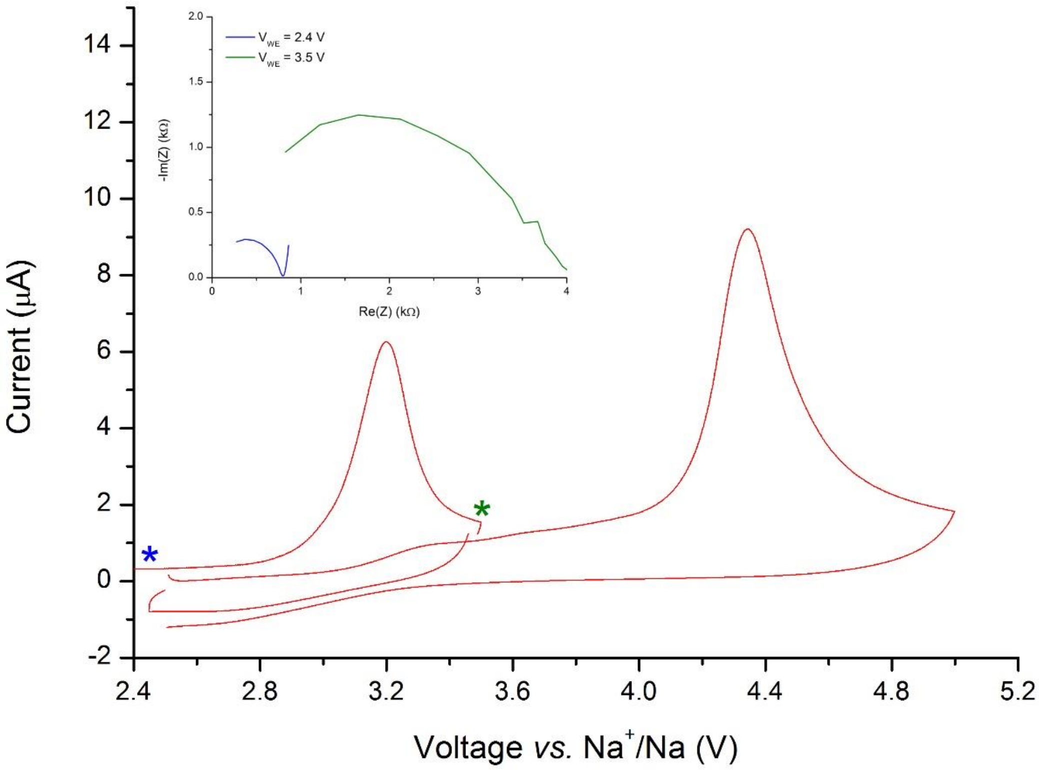

Figure S7: Evolution of the conductivity of $Na_{0.33}(CB_{11}H_{12})_{0.33}$($m$-$C_2B_{10}H_{12})_{0.66}$ before and after the peak at 3 V *vs.* Na^+/Na.

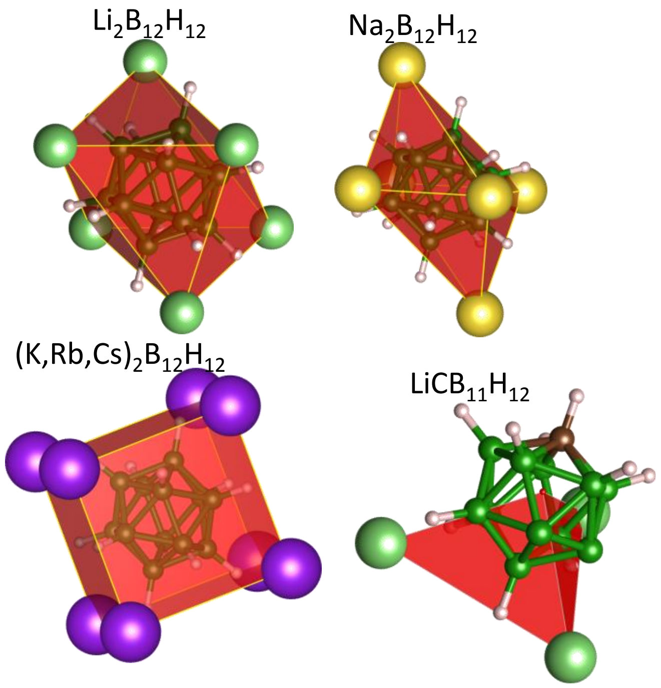

Figure S8: Coordination of anions in selected *closo*-hydridoborates.

[1] P. von Ragué Schleyer and K. Najafian, Stability and Three-Dimensional Aromaticity of *closo*-Monocarbaborane Anions, $CB_{n-1}H_n^-$, and *closo*-Dicarboranes, $C_2B_{n-2}H_n$. *Inorg. Chem.* **1998**, *37*(14), 3454-3470.
